# Supplementary material for: Building a public health workforce for a university campus during a pandemic using a practicum framework: Design and outcomes
Source: PLoS One. 2022 Jul 21;17(7):e0270488. doi: 10.1371/journal.pone.0270488 (PMC9302791; doi:10.1371/journal.pone.0270488)
Supplement: Attachment — Submitted filename: PlosOneReponse_032322FIN.docx [file pone.0270488.s001.docx]

**1. Abstract need to be restructured.**

Thank you for pointing this out. As suggested, the Abstract has been restructured.

**2. Introduction lacks contribution and structure of what the other section will provide.**

Text in the Introduction was revised with clarification,

Using a practicum framework, a West Coast university with an undergraduate enrollment of 31,162 students, developed a public health workforce of undergraduate peer educators to address its reopening plans and meet its COVID-19 health promotion mandate. In this paper, we describe the use and outcomes of a practicum framework to create a university-based public health workforce to limit the spread of COVID-19 on a university campus. We address two questions: (1) Using a practicum framework, what are important considerations in designing and building a workforce for a university campus? and (2) What are the benefits to the workforce in terms of public health education and professional growth? This paper contributes to filling the gap in the literature about how a practicum framework can be used on a large scale as an effective learning tool in an urgent public health crisis.

**3. Abstract should reflect the background knowledge on the problem addressed need to be added.**

As suggested, a Background section was added. It reads:

Background: The reopening of college and university campuses was seen as presenting a high risk for transmission of COVID-19. Thus, these institutions faced with a new public health challenge never heretofore faced on this scale. To magnify the problem, they needed to rapidly develop and implement re-opening plans in an environment filled with uncertainty and for a population that was significantly less likely to observe COVID-19 mitigation behaviors. In response, within three weeks of opening, as part of its COVID-19 public health strategy, a West Coast university created and trained a public health workforce comprised of 282 undergraduates tasked with encouraging compliance with COVID-19 mitigating healthy behaviors.

**4. Abstract should reflect the wide range of applications and its possible solutions need to be added.**

As suggested, text has been added:

These findings demonstrate an effective way of rapidly addressing public health concerns that allowed for on the job training and opportunities for young adults to learn and grow.

**5. Abstract should reflect the problem addressed need to be justified with more details.**

As suggested, additional text

**Background:** The reopening of college and university campuses was seen as presenting a high risk for transmission of COVID-19. Thus, these institutions faced with a new public health challenge never heretofore faced on this scale. To magnify the problem, they needed to rapidly develop and implement re-opening plans in an environment filled with uncertainty and for a population that was significantly less likely to observe COVID-19 mitigation behaviors. In response, within three weeks of opening, as part of its COVID-19 public health strategy, a West Coast university created and trained a public health workforce comprised of 282 undergraduates tasked with encouraging compliance with COVID-19 mitigating healthy behaviors.

**6. In Introduction section, the drawbacks of each conventional technique should be described clearly.**

As suggested, text was revised and added:

But, to provide effective education, peers must be prepared with knowledge and communication skills. In public health, practicum structures are used as a teaching vehicle. Practicums present students opportunities to apply public health knowledge and skills in practice [12]. Undergraduate practicum projects have been shown to be highly effective to train students for public health work [13]. Thus, the practicum is considered a capstone in an undergraduate student’s public health experience [14]. This assumes that the practicum is being offered within the context of a public health major, raising the question of the effectiveness of a practicum framework to introduce and teach public health to students who are not majoring in public health. Furthermore, there is a paucity of literature on the use of the practicum model on a large scale to address a community’s public health needs that a pandemic demands. These demands include continuously keeping up to date with quickly changing scientific knowledge, public health policies, and educating a potentially frustrated public while creating a positive learning experience.

**7. Introduction section can be extended to add the issues with respect to existing work**

As suggested, text was revised and added:

But, to provide effective education, peers must be prepared with knowledge and communication skills. In public health, practicum structures are used as a teaching vehicle. Practicums present students opportunities to apply public health knowledge and skills in practice [12]. Undergraduate practicum projects have been shown to be highly effective to train students for public health work [13]. Thus, the practicum is considered a capstone in an undergraduate student’s public health experience [14]. This assumes that the practicum is being offered within the context of a public health major, raising the question of the effectiveness of a practicum framework to introduce and teach public health to students who are not majoring in public health. Furthermore, there is a paucity of literature on the use of the practicum model on a large scale to address a community’s public health needs that a pandemic demands. These demands include continuously keeping up to date with quickly changing scientific knowledge, public health policies, and educating a potentially frustrated public while creating a positive learning experience.

**8. What is the motivation of the proposed work?**

As suggested, text was added:

This paper contributes to filling the gap in the literature about how a practicum framework can be used on a large scale as an effective learning tool in an urgent public health crisis.

It also created an infrastructure to expeditiously educate and prepare a large workforce while providing ongoing education about new, salient, and rapidly changing scientific knowledge and public health policies to a potentially confused and frustrated populace.

**9. Literature review techniques have to be strengthened by including the issues in the current system and how the author proposes to overcome the same.**

The explanation was added:

But, to provide effective education, peers must be prepared with knowledge and communication skills. In public health, practicum structures are used as a teaching vehicle. Practicums present students opportunities to apply public health knowledge and skills in practice [12]. Undergraduate practicum projects have been shown to be highly effective to train students for public health work [13]. Thus, the practicum is considered a capstone in an undergraduate student’s public health experience [14]. This assumes that the practicum is being offered within the context of a public health major, raising the question of the effectiveness of a practicum framework to introduce and teach public health to students who are not majoring in public health. Furthermore, there is a paucity of literature on the use of the practicum model on a large scale to address a community’s public health needs that a pandemic demands. These demands include continuously keeping up to date with quickly changing scientific knowledge, public health policies, and educating a potentially frustrated public while creating a positive learning experience.

This paper contributes to filling the gap in the literature about how a practicum framework can be used on a large scale as an effective learning tool in an urgent public health crisis.

**10. Research gaps, objectives of the proposed work should be clearly justified.**

The explanation was added:

But, to provide effective education, peers must be prepared with knowledge and communication skills. In public health, practicum structures are used as a teaching vehicle. Practicums present students opportunities to apply public health knowledge and skills in practice [12]. Undergraduate practicum projects have been shown to be highly effective to train students for public health work [13]. Thus, the practicum is considered a capstone in an undergraduate student’s public health experience [14]. This assumes that the practicum is being offered within the context of a public health major, raising the question of the effectiveness of a practicum framework to introduce and teach public health to students who are not majoring in public health. Furthermore, there is a paucity of literature on the use of the practicum model on a large scale to address a community’s public health needs that a pandemic demands. These demands include continuously keeping up to date with quickly changing scientific knowledge, public health policies, and educating a potentially frustrated public while creating a positive learning experience.

This paper contributes to filling the gap in the literature about how a practicum framework can be used on a large scale as an effective learning tool in an urgent public health crisis.

**11. The conclusion should state scope for future work.**

As suggested, text was added to suggest further work,

This approach may not be fully replicable for universities without a public health educational program. But, they may be able to find other related fields from which to draw. Future work could pursue innovative ways of replicating a similar workforce through academic and governmental partnerships.

**12. Authors should include some graphs, flowcharts for better presentation of work**

As suggested, the flowchart was moved from supplemental material to the manuscript.

**Reviewer #2:**

**1. Are there any patterns across academic majors in terms of STEM vs. non-STEM students?**

We examine whether the differences in satisfaction by STEM. There were none. We included these analyses in Table 3 and the text,

Of the PHA respondents, 98.8% rated their satisfaction with the PHA role (Table 3). The majority indicated they were satisfied with their role (95.2%, n = 238). Few were dissatisfied with their role either (4.8%, n = 12. In addition, 96.8% (n = 242) were satisfied the educational value of the program and 94.0% (n. =235) were satisfied with the value for their professional interests. There were no significant differences by either gender or STEM/Non-STEM major. Furthermore, none of the PHAs developed COVID-19 as a result of their role.

**2. Are there statistically significant differences in perceptions due to their gender?**

We examine whether there were differences in satisfaction by gender. There were none. We included these analysis in Table 3 and the text,

Of the PHA respondents, 98.8% rated their satisfaction with the PHA role (Table 3). The majority indicated they were satisfied with their role (95.2%, n = 238). Few were dissatisfied with their role either (4.8%, n = 12. In addition, 96.8% (n = 242) were satisfied the educational value of the program and 94.0% (n. =235) were satisfied with the value for their professional interests. There were no significant differences by either gender or STEM/Non-STEM major. Furthermore, none of the PHAs developed COVID-19 as a result of their role.

**3. Additional factors that the authors could have considered include compensation, assignment of trivial work, competitive co-interns, and work overload.**

All were paid the same rate. The APHAs were assigned the same roles. We were careful to ensure that their roles involved public health policy education and the role did not deviate from this function. Most worked 10 hours/week.
